# Work as the missing link in Europe’s Climate and Health Strategic Research Agenda

**DOI:** 10.5271/sjweh.4328

**Published:** 2026-11-01

**Authors:** Maria Albin, Michelle C Turner, Sara Svensson, Mary Njoroge, Susana Viegas, Christopher Mathieu, Neil Pearce, Alex Burdorf, Kurt Straif, Karin Broberg, Manolis Kogevinas

**Affiliations:** 1 Institute of Environmental Medicine, Karolinska Institutet, Stockholm, Sweden.; 2 Division of Occupational and Environmental Medicine, Lund University, Lund, Sweden.; 3 Barcelona Institute for Global Health (ISGlobal), Barcelona, Spain.; 4 Universitat Pompeu Fabra (UPF), Barcelona, Spain.; 5 Consortium for Biomedical Research in Epidemiology & Public Health (CIBER Epidemiología y Salud Pública – CIBERESP), Madrid, Spain.; 6 School of Education, Humanities and Social Sciences, Halmstad University, Halmstad, Sweden.; 7 Department of Medical Statistics, London School of Hygiene and Tropical Medicine, London, United Kingdom.; 8 NOVA National School of Public Health, Public Health Research Centre, Comprehensive Health Research Center, CHRC, REAL, CCAL, NOVA University Lisbon, Lisbon, Portugal.; 9 Department of Sociology, Lund University, Lund, Sweden.; 10 Department of Public Health, Erasmus University Medical Center, Rotterdam, the Netherlands.

**Keywords:** climate change, environmental policy, green transition, occupational health, occupational safety, working condition

## Abstract

**Objective:**

The aim of this Discussion Paper is to examine the integration of occupational health within the European Strategic Research and Innovation Agenda on Health and Climate Change (SRIAHCC) and identify critical gaps relevant to worker health, equity, and the European Green Deal.

**Methods:**

We analyze the SRIAHCC in relation to related EU policy documents on occupational safety and health, climate change and the green transition, and other current scientific evidence. In addition, we performed an original survey and workshop consultations with key stakeholders in the field and conducted a Delphi-inspired consultation round within the EU INTERCAMBIO project.

**Results:**

While the SRIAHCC appropriately frames climate change as a public health emergency, occupational health is not systematically addressed either as a structural determinant of climate vulnerability or as a core domain of adaptation and mitigation research. Direct and indirect occupational exposures including heat stress, extreme weather events, air pollution, and emerging risks in relevant sectors, eg, decarbonization and circular economy, receive limited attention beyond the healthcare sector. Psychosocial impacts, inequalities affecting migrant and precarious workers, and productivity and income consequences are insufficiently addressed. Furthermore, the agenda lacks a clear implementation framework for workplace adaptation and harmonized occupational-climate monitoring systems.

**Conclusions:**

Without explicit integration of work and working conditions into the SRIAHCC, Europe’s climate and health strategy likely underestimates the true health costs of climate change and the green transition. Embedding occupational health as a core pillar of the SRIAHCC is essential to ensure coherent EU governance, equitable worker protection, and a resilient, sustainable transition.

The European Commission’s 2025 proposal to double funding for its research and innovation framework programme Horizon Europe, marks a critical moment for defining Europe’s climate and health research. The current Strategic Research and Innovation Agenda on Climate Change and Health (SRIAHCC) (1) represents a key instrument for shaping future research investments on the topic in joint European research funding while also setting priorities for national funding within the EU.

Since 2019, the European Green Deal has provided the overarching framework for aligning economic transformation with climate mitigation, environmental protection, and health. Importantly, the Green Deal acknowledges the need for the transition to be fair and inclusive by explicitly committing to a transition that “leaves no one behind”. This ambition aligns with the European Pillar of Social Rights and the EU Strategic Framework on Occupational Safety and Health (2021–2027), both of which emphasize fair working conditions and worker protection as cornerstones of social Europe.

Initiatives such as the Zero Pollution Action Plan and the Green Industrial Deal underline the ambition to decarbonize Europe’s economy while safeguarding health and ecosystems. However, concerns have emerged that competitiveness and industrial priorities risk overshadowing social and health dimensions, particularly those related to work and working conditions. Also, current resetting of international trade and energy markets and the political landscapes within major member countries have facilitated a renewed trend towards deregulation, undercutting, eg, decarbonization (2).

Other policy documents of relevance include the Foresight Report (3), which reflects the growing use of scenario-building in European policymaking. This report frames climate change as a major global “mega-trend” shaping a broader polycrisis context, alongside economic, migration, and public health crises, and calls for reimagining education and work in response to technological change, demographic shifts, and future disruptions. These concerns are echoed in the Occupational Safety and Health Pulse 2025 (4), which reports that three in ten workers are worried about the impacts of climate change on their safety and health at work.

Workers in climate-exposed sectors are disproportionately male, poorly educated, seasonal, migrant, or self-employed, and often lack adequate representation and protection (5). Strengthening resilience therefore requires systemic approaches that combine adaptive occupational safety and health regulation, high job quality standards, skills development, and sector-specific guidance. In December 2025, the European Commission’s Advisory Committee on Safety and Health at Work issued an opinion on climate change and heat at work (6), recommending EU-level action to improve strategies for assessing occupational heat exposure and to adopt evidence-based preventive interventions, including hydration, work organization, and heat acclimatization. Another opinion further emphasized the need for practical, sector-specific guidance (7).

There is already solid evidence showing that climate change is reshaping work and working conditions across Europe and globally. According to current ILO estimates (8), at least 2.41 billion workers are exposed to excessive heat every year globally, corresponding to >70% of all workers. Rising temperatures, more frequent heatwaves, air pollution, extreme weather events, and shifting patterns of infectious disease exposure all directly and indirectly affect workers’ health, safety, and productivity. Outdoor workers, those performing physically demanding tasks and workers in poorly climate-controlled indoor environments are particularly vulnerable (8–13).

The interface between climate change, work and health has been most extensively documented for occupational heat exposure. Evidence increasingly links occupational heat exposure not only to acute outcomes such as heat exhaustion and injury, but also to chronic effects including kidney, cardiovascular, neurological disease, and mental health disorders (14). Key recommended interventions include heat action plans, use of new technologies for risk assessment (warning, surveillance) and mitigation (eg, cooling vests), and social protection, with a need for implementation studies on a sector and regional level (10).

In addition to the effects of heat, climate-related hazards such as wildfire smoke, flooding, and extreme precipitation events disproportionately affect workers involved in emergency response, recovery, construction, and agriculture (13, 15). These risks are often compounded by precarious employment, migration status, and limited access to occupational health services. At the same time, productivity losses and income reductions associated with climate exposures risk reinforcing existing social and health inequalities (16). Thus, it is likely that the trend during the last decade of increasing social inequalities in mental and physical health within member states, as reflected in the European Social Survey (17), will continue to widen.

Within this evolving landscape of policy and evidence, the SRIAHCC will strongly influence European and member state funding decisions by organizing priorities around the impacts of climate change on health, as well as mitigation, resilience, and adaptation. European funding is of fundamental importance to close critical knowledge gaps in this area also in a wider context: firstly because independent funding is key for a research field with strong conflicting interests, secondly because US federal funding for climate and health research has been halted including rescinding core scientific positions, and ending programs at the National Institutes of Health (18), thus increasing the global relative importance of the European contribution.

The focus of this Discussion Paper is the extent to which occupational health is integrated into this agenda and what long-term implications this has for Europe’s capacity to protect workers and ensure a just green transition and resilient societies. These factors are also structural enablers of sustainable European competitiveness and economic growth.

This paper focuses on the SRIAHCC because of its strategic role in guiding future European research funding. To contextualize the analysis, we provide some examples from other relevant European policy documents addressing occupational safety and health, climate adaptation and the green transition, together with other recent scientific literature. We also performed a survey and workshop consultations with stakeholders within the framework of the EU INTERCAMBIO project (19) – including researchers, social scientists and social partners – and conducted a Delphi-inspired (20) consultation of the multi-disciplinary expertise found among the INTERCAMBIO project members. The survey (21) was based on purposive sampling of researcher and non-researcher stakeholders (eg, experts at agencies, social partners on the European and national levels; 91 respondents, 18% response rate) and supplemented with two interactive workshops (one face-to-face and one virtual; total of 70 participants) in 2025 (see, eg 22,). Based on a first overall assessment of the SRIAHCC and the stakeholder consultations, experts within the INTERCAMBIO project contributed a more detailed assessment within their respective areas of expertise, and this was iteratively condensed into the present paper. The conceptual framework we follow regarding the Climate–Work–Health Interface is shown in figure 1.

**Figure 1:**
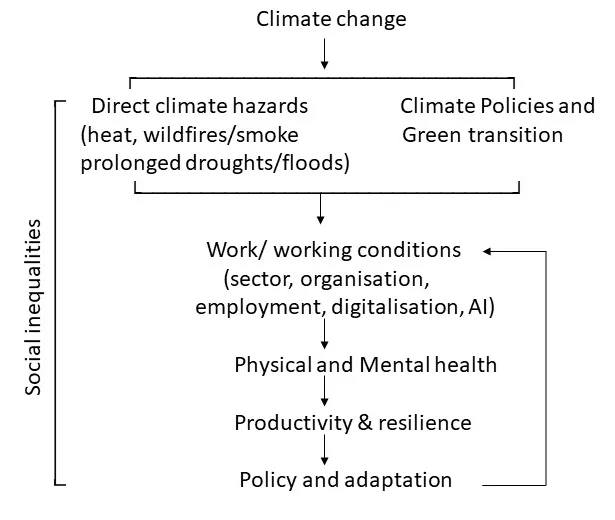
Conceptual framework used in this paper. The framework was developed through multidisciplinary discussions among researchers participating in the INTERCAMBIO project and stakeholder consultations involving occupational health researchers, social scientists, policy experts and social partners. It guided our interpretation of the SRIAHCC.

Throughout the paper, *work and working conditions* refers to what could be said to be the broadest interpretation of the concept, ie, encompassing employment relations, work organization and occupational environments. *Occupational safety and health (OSH)* refers to policies and practices addressing prevention of occupational injuries and diseases. *Occupational health* refers primarily to the health consequences of work and occupational exposures, whereas *worker health* is used in its broadest sense, including occupational and non-occupational determinants affecting people in employment.

Briefly, main components of the SRIAHCC include as cross-cutting areas inter-and transdisciplinary collaboration; climate-driven health inequalities; monitoring models, tools and technologies; data- and research infrastructures. The highlighted research gaps include: direct effects of climate change on health; interactions with environmental enablers of health; mitigation and resilience in the health sector, implementation research to bridge the gap between research and action (1).

## Gaps in the integration of work and a just transition

A key gap in the SRIAHCC is its limited attention to the direct interaction between climate change, mitigation strategies and work. Beyond broad references to OSH, mitigation is largely discussed in relation to the healthcare sector and associated health co-benefits. Yet, the primary pathway for climate mitigation – the green transition – directly reshapes work across multiple sectors, including energy, chemical industries, construction, transport, and waste management. Encompassing renewable energy transition, circular economy processes, and new work practices, the green transition influences both mental and physical health through work. These effects are strongly shaped by how work is organized. Exposures and preventive actions depend on managerial decisions, worker knowledge, and employment relations. Evidence from recycling and other green sectors show that high turnover, low wages, and contingent employment undermine both occupational safety and mental well-being (23, 24).

Emerging evidence also points to hazardous exposures in sectors central to the green transition. Workers in lithium-ion battery production experienced elevated exposure to nickel and cobalt and increased risk of occupational skin disease (25, 26) as do workers due to epoxy exposure in the wind turbine industry (27). Renewable fuels may produce toxic effects comparable to diesel exhaust (28), while metal recycling involves exposure to complex mixtures of metals with known or unknown toxicity (29). Musculoskeletal disorders and occupational injuries are common (30, 31), whereas psychosocial risks and mental health outcomes remain understudied. An emerging issue is potential downstream occupational exposures associated with reuse and recycling of eg, construction and plastic waste, into new products, where legacy contaminants embedded in products and materials (eg, asbestos, toxic metals, and brominated flame retardants) may be reintroduced into workplaces.

Europe’s green transition relies heavily on global supply chains for critical minerals such as lithium, cobalt, nickel and rare earth elements. Occupational risks associated with extraction, processing and recycling are frequently transferred to low- and middle-income countries. A genuinely just transition therefore requires occupational health considerations across the entire value chain, rather than only within European workplaces.

The green transition is closely intertwined with digitalization, creating synergies but also contradictions and unintended consequences for work organization and working conditions. Digitalization and use of AI may intensify work pace, redundancies, increase surveillance, and alter autonomy, with implications for mental health and work-related stress. Evidence suggests that worker involvement can mitigate some of these risks (32). Global scenarios for 2030 on job creation and job loss by ILO (33) indicate substantial labor market effects of the transition to a circular economy. Worldwide, an estimated 71 million jobs may disappear, while approximately 78 million new jobs could be created, resulting in a modest net employment gain. However, this transition will require significant workforce adaptation: around 22 million workers are expected to need to move into new occupations, and approximately 49 million will require substantial reskilling to remain in their current occupational fields. The estimated global labor market effect of the energy transition was less, an estimated potential loss of 7 million jobs but at the same time growth of 25 million jobs. The major changes were foreseen within medium-skilled occupations both for job loss and gains.

The SRIAHCC highlights the strain on the healthcare sector from eg, increasing health demands and emergencies caused by climate change, the need for sustainable healthcare delivery, as well as the substantial carbon footprint of healthcare. Mental health impacts, however, are not just sectoral (eg, healthcare), but also structural, including precarity, re-skilling anxiety, and employment transitions. Even within the healthcare sector, the analysis of how climate change and sustainability measures affect healthcare workers themselves is less developed (19). Emerging evidence suggests that sustainable work practices, ie, aiming at reducing the carbon footprint, may both alleviate (34) and exacerbate psychosocial stress (35), depending on organizational context, workload, and resources.

Climate-related mental health concerns are also gaining clinical prominence, but are under-researched and prioritized. Healthcare and first-responder workers increasingly face “vicarious” indirect climate impacts through secondary trauma associated with treating populations affected by climate-related disasters (36). Mental health clinicians face growing challenges in addressing climate-related mental health problems which they report being ill-prepared to deal with (37). These dynamics underscore the need to integrate occupational mental health perspectives into healthcare sustainability strategies.

Achieving both health and climate goals requires deliberately integrating health-promoting and climate-beneficial work practices beyond healthcare, particularly in core transition sectors. Where occupational health and climate objectives do not naturally align, compatibility must be actively fostered through considered, optimal rather than expedient, trade-offs at industry, organizational, and workplace levels. Strengthening worker voice, autonomy and the presence of organized labor has been shown to support both improved occupational health and environmentally sustainable workplace practices (38–40). Tailored interventions, co-designed with affected workers, are particularly important for migrant and precarious populations who face compounded vulnerabilities (41).

Finally, a multi-logic approach is needed in which health, climate, and work-environment risks are jointly identified, prioritized and managed from the workplace to the societal levels. Such an approach supports multiple forms of sustainability – health, environmental, social and economic – and situates work within its broader societal and planetary context.

## Occupational health dimensions insufficiently represented in the SRIAHCC

Several cross-cutting additional dimensions are insufficiently represented and integrated into the SRIAHCC (table 1), and are briefly outlined below.

**Table 1:** Summary of which occupational health dimensions were assessed as fully, partially, negligibly covered/acknowledged, or absent from the SRIAHCC agenda.

Occupational dimension	Coverage in SRIAHCC	Rationale (page number in SRIAHCC)
Heat	Partially covered/acknowledged	Short- and long-term health effects on workers critical area for research and innovation (p 24).
Extreme events	Partially covered/acknowledged	Emergency workers briefly mentioned (p 24), resilience in health sector a priority (p 35).
Climate mitigation and worker health	Negligibly covered/acknowledged	Only covered for healthcare sector (pp 32–34). Transition to circular economy or fossil-free energy not covered.
Mental health	Negligibly covered/acknowledged	Vulnerable and precarious workers briefly mentioned (p 27).
Occupational inequalities	Negligibly covered/acknowledged	Climate change exacerbation of inequalities is a cross-cutting theme and mentions workers (p 16), but does not explicitly take this further into critical areas for research and innovation.
Sector-specific interventions	Negligibly covered/acknowledged	Not covered outside healthcare sector.
Monitoring systems	Negligibly covered/acknowledged	Need for good quality exposure and health data related to climate change is mentioned (p 24), but but does not include climate mitigation and is not explicitly taken forward to section on Research data and infrastructure (pp 19–22).
Implementation research	Negligibly covered/acknowledged	Only healthcare sector (pp 36–37).

*Addressing workplace and sector needs.* The SRIAHCC does not sufficiently address the urgent need for sector- and workplace-specific evidence-based adaptation guidance. Implementation- and participatory-oriented research is required to support risk perception and interventions and evaluate key risk reduction strategies, including heat stress assessment, acclimatization of new and returning workers, hydration practices, adaptation of personal protective equipment, and work organization measures such as self-pacing, task rotation, and working-time adjustments. Adaptation strategies must also integrate workforce vulnerability related to environmental conditions (eg, heat stress during commuting and at home) and individual factors such as age, and chronic diseases.

*Knowledge and training gaps.* Although knowledge translation is identified as a cross-cutting theme, research needs on training and capacity building outside the healthcare sector remain underdeveloped. While some training resources on climate-related occupational risks, particularly heat stress, are increasingly available for workers and employers, rapidly evolving exposures and technologies associated with climate change and the green transition require agile, accessible, and tailored knowledge transfer systems, as well as stronger interaction between technology developers and occupational health research (“safe and sustainable by design”).

*Economic and productivity aspects.* Although the SRIAHCC focuses mainly on health, productivity losses and economic impacts are closely linked to socioeconomic inequalities. Reliable data on income loss, reduced work capacity, and compensatory increases in working time, stratified by socioeconomic position, sex, region, and sector, are essential to inform timely and equitable policy responses. Such efforts may also strengthen economic competitiveness by supporting sustainable production, technological leadership, and resilient economic systems in the face of climate change.

*Mental health and psychosocial dimensions.* Climate change and mitigation actions affect worker mental health and well-being through multiple pathways and at multiple levels, reaching from gross trends in changing employment relations and work organization associated with the dual green and digital transitions, to direct effects in specific occupational sectors (eg, agriculture, construction, and first responders to extreme events). Research should prioritize strategies that enhance resilience, worker empowerment, and worker participation in workplace and transition-related decision-making, while accounting for major labor market shifts, job loss/creation, and for re- and upskilling needs.

*Indicators and monitoring.* As indicated in the SRIAHCC, by the Advisory Committee on Safety and Health at Work (6), and in other reports and suggested agendas (13, 42) mitigation and regulatory policies, require harmonized and robust indicators for monitoring exposures and health outcomes. These systems should integrate occupational and environmental data in exposure-response surveillance frameworks, drawing on existing data sources and research platforms and supporting early detection of emerging risks. Without harmonized occupational-climate surveillance systems, the EU risks systematically underestimating the true health costs of climate change and mitigation actions.

*Global dimension.* Europe is not isolated. European climate policy has transnational occupational implications, through green supply chains, imported materials (eg, lithium, cobalt) and exported materials (waste) and occupational risks externalized to non-EU countries. The agenda does not sufficiently cover these issues.

## Build on strong elements of the SRIAHCC

The above discussion has focused on gaps and the need for additional dimensions to respond to the challenges to worker health and well-being from climate change and mitigation action. There are also several calls for new and extended approaches in the SRIAHCC that are promising and may be strong pillars in research addressing these challenges. Occupational research (in a broad sense) should be well equipped to take this on. These approaches/elements include crosscutting themes such as increased *inter- and transdisciplinarity* in research collaborations, closer attention to activities on *citizen behavior, education and empowerment*, and greater emphasis on understanding and reducing *climate-driven health inequalities* for fair and people-centered action. Also needs to underpin science are identified including improvements in *data and research infrastructures*, and more adequate *models, tools and technologies* for monitoring and prevention.

## Implications for a future occupational health research agenda for Europe

The various evidence reviewed here supports the inclusion of occupational health as a core pillar of Europe’s climate and health research agenda. Stakeholders (eg, researchers, agencies, social partners) participating in our survey and workshops consistently prioritized heat stress, extreme weather events, mental health, and emerging risks in green sectors, alongside a strong emphasis on vulnerable worker groups and implementation-focused research. Social partners consulted also highlighted a willingness to engage with research.

## Recommendations for future research agendas

This paper is in informed by consultations with a purposive sample of key stakeholders and an iterative process between multi-disciplinary experts including examination of key European policy documents and recent scientific evidence and reflects expert judgement. The purpose is not to provide a comprehensive assessment of all climate-health policies but to identify major gaps relevant to occupational health and stimulate further policy and research development.

We identify five initial priorities that could strengthen future revisions of the SRIAHCC (box 1). There is conceptual overlap between our recommendations and those proposed by Frank et al (9), which is expected given that both seek to advance occupational health research. However, our recommendations were developed for a different purpose: to inform the revision of the SRIAHCC. Consequently, they specifically prioritize the integration of occupational health into climate adaptation and mitigation research, harmonized occupational–climate surveillance, implementation science, sector-specific adaptation, and the just transition, rather than proposing a general occupational health research agenda.

Box 1 **Five Priorities for Integrating Occupational Health into the SRIAHCC**

1. Include work as a structural determinant of climate vulnerability. Recognize work and working conditions as key pathways through which climate change and climate policies affect health, particularly for workers in climate-exposed, precarious and rapidly transforming occupations.

2. Fund sector-specific adaptation research. Support intervention and implementation research to develop, evaluate and scale workplace adaptation strategies for sectors such as agriculture, construction, transport, manufacturing, healthcare, and the green economy.

3. Develop harmonized occupational-climate monitoring systems. Create integrated surveillance systems that combine occupational exposure, health and climate data to monitor emerging risks, develop harmonized occupational-climate indicators, evaluate interventions and guide policy across Europe.

4. Address psychosocial, inequality and just transition dimensions. Strengthen research on mental health, work organization, labor market transitions and the unequal impacts of climate change and mitigation policies on vulnerable workers, including migrants, temporary workers and older workers.

5. Invest in implementation science science and policy translation. Move beyond hazard identification by evaluating effective workplace interventions, participatory approaches, governance mechanisms and policies that protect worker health while supporting climate adaptation and mitigation.

As earlier done within the Health Environment Research Agenda for Europe (HERA) consortium (43), an updated European research agenda on occupational health is currently being developed within the EU INTERCAMBIO project and will provides a concrete framework for advancing this integration (19). It will build on continued work in hazard identification, including, eg, cancer risk associated with climate change (44), while placing greater emphasis on systemic implementation, emphasizing the need to embed occupational health into climate, energy, and industrial policies. Key priorities may likely include addressing the climate-work interface, preventing new risks in decarbonization and circular economy activities, and tackling demographic inequalities.

Importantly, a transformation in research approaches is also needed, with greater emphasis on intervention studies, implementation science, and science-policy translation. Using existing advanced data infrastructures, including sensor technologies and AI-based monitoring, offers opportunities to move from reactive to preventive occupational health strategies.

If occupational health is not included when setting and updating the research agenda, it may be structurally excluded from the next research and funding cycle, not only at the EU level, but also in member states since the ambition of the Commission is that the SRIAHCC should also guide national funding. This would clearly have serious consequences given Europe’s aging workforce and rapidly increasing heat exposure, a combination of uncontrolled exposures and labor shortages in green sectors, aggravated by the context of increasing political push for deregulation and a one-dimensional competitiveness narrative (2), overshadowing worker protection.

## Conclusions: strengthening Europe’s climate and health strategy

Occupational health remains insufficiently integrated into the current SRIAHCC. Without explicit integration of work and working conditions into the SRIAHCC, there is a risk that climate mitigation and adaptation efforts will inadvertently undermine worker health, exacerbate inequalities, and weaken societal resilience.

Aligning the SRIAHCC with an expanded occupational health research agenda is therefore not only a matter of worker protection, but also a prerequisite for a successful and just green transition. This requires strategic engagement to position occupational health at the intersection of climate, health, and social policy; the development of new alliances across disciplines and sectors; and sustained investment in research that supports implementation, participation, and equity.

Renewed versions of the SRIAHCC should explicitly: (i) recognize work as a structural determinant of climate vulnerability; (ii) include occupational health as a dedicated research priority in climate change and health; (iii) support sector-specific climate adaptation research; (iv) develop integrated occupational-climate surveillance systems; (v) strengthen climate change-related implementation science and intervention studies; and (vi) incorporate the impacts of the green transition on work environment, work organization, inequalities and mental health.

Maintaining occupational health at the periphery of the SRIAHCC risks undermining the EU’s objectives of a just and resilient green transition. Work is not only affected by climate change, it is a central pathway through which mitigation policies reshape exposures, redistribute risks, and potentially widen or reduce inequalities. If occupational health is not included at this stage of research agenda-setting, it risks structural exclusion from the next cycle of European research, funding and policy development. This would weaken the evidence-base and coherence between the European Green Deal, the EU Strategic Framework on OSH, and broader commitments to social equity. A renewed research agenda should complement continued efforts in hazard identification with stronger emphasis on implementation science, participatory approaches, and harmonized occupational-climate monitoring systems. Integrating worker voice, sector-specific adaptation strategies, and attention to vulnerable groups is not only a matter of protection but of inclusion and democratic legitimacy, thus responding also to wider primary concerns of the Commission. Europe has the scientific capacity and institutional infrastructure to lead globally in climate-responsive occupational health. The question is whether this opportunity will be seized while the strategic agenda is further developed.
